# The Effects of Inorganic Nitrogen form and CO_2_ Concentration on Wheat Yield and Nutrient Accumulation and Distribution

**DOI:** 10.3389/fpls.2012.00195

**Published:** 2012-09-03

**Authors:** Eli Carlisle, Samuel Myers, Victor Raboy, Arnold Bloom

**Affiliations:** ^1^Department of Plant Sciences, University of CaliforniaDavis, CA, USA; ^2^Harvard School of Public Health, Harvard UniversityBoston, MA, USA; ^3^Agricultural Research Service, United States Department of AgricultureAberdeen, ID, USA

**Keywords:** climate change, wheat, ammonium, nitrate, nutrients, grain, phytate, CO_2_

## Abstract

Inorganic N is available to plants from the soil as ammonium $\left({\text{NH}}_{4}^{+}\right)$ and nitrate $\left({\text{NO}}_{3}^{-}\right)$. We studied how wheat grown hydroponically to senescence in controlled environmental chambers is affected by N form (${\text{NH}}_{4}^{+}$ vs. ${\text{NO}}_{\text{3}}^{-}$) and CO_2_ concentration (“subambient,” “ambient,” and “elevated”) in terms of biomass, yield, and nutrient accumulation and partitioning. Wheat supplied with ${\text{NH}}_{4}^{+}$ as a sole N source had the strongest response to CO_2_ concentration. Plants exposed to subambient and ambient CO_2_ concentrations typically had the greatest biomass and nutrient accumulation under both N forms. In general ${\text{NH}}_{4}^{+}$-supplied plants had higher concentrations of total N, P, K, S, Ca, Zn, Fe, and Cu, while ${\text{NO}}_{\text{3}}^{-}$-supplied plants had higher concentrations of Mg, B, Mn, and ${\text{NO}}_{\text{3}}^{-}\text{ - N}.$ ${\text{NH}}_{4}^{+}$-supplied plants contained amounts of phytate similar to ${\text{NO}}_{\text{3}}^{-}$-supplied plants but had higher bioavailable Zn, which could have consequences for human health. ${\text{NH}}_{4}^{+}$-supplied plants allocated more nutrients and biomass to aboveground tissues whereas ${\text{NO}}_{\text{3}}^{+}$-supplied plants allocated more nutrients to the roots. The two inorganic nitrogen forms influenced plant growth and nutrient status so distinctly that they should be treated as separate nutrients. Moreover, plant growth and nutrient status varied in a non-linear manner with atmospheric CO_2_ concentration.

## Introduction

Nitrogen (N) is the mineral element that most often limits plant growth and primary productivity in natural and agricultural systems. Plants usually acquire N from the soil in the forms of ammonium (${\text{NH}}_{4}^{+}$) and nitrate (${\text{NO}}_{\text{3}}^{-}$) and management of these forms is vital to agriculture. Wheat can utilize either form alone (Wang and Below, 1992), but mixed N nutrition (e.g., NH_4_NO_3_) typically produces the best grain yields and quality in hydroponically grown (Gentry et al., 1989; Heberer and Below, 1989; Wang and Below, 1995) and field-grown plants (Bock, 1987; Camberato and Bock, 1990).

Ammonium and nitrate affect crops differently when either is supplied as the sole N source (Bloom, 1997). Ammonium requires less energy to assimilate into organic compounds (Bloom, 1997), but can prove toxic if it accumulates to high concentrations within plant tissues (Cox and Reisenauer, 1973; Goyal and Huffaker, 1984). Nitrate is generally the predominant form available in aerated, temperate agricultural soils (Haynes, 1986; Bloom, 1997), and may accumulate within plant tissues to high concentrations without toxicity (Goyal and Huffaker, 1984). In wheat, the N form supplied has been found to influence many physiological parameters profoundly including biomass (Wang and Below, 1995, 1996, 1998; Bloom et al., 2002), leaf area (Bloom et al., 2002), tillering (Chen et al., 1998), seed mass (Wang and Below, 1995), protein content (Wang and Below, 1995), and mineral nutrient acquisition and distribution (Gashaw and Mugwira, 1981; Wang and Below, 1998), although such differences can vary among cultivars (Gashaw and Mugwira, 1981; Wang and Below, 1995).

The presence of ${\text{NH}}_{4}^{+}$, as either a sole N source or in mixed N nutrition, increased organic N concentration in shoots, roots, and grain and decreased partitioning of dry matter to the roots in wheat (Wang and Below, 1995). Decreased cation uptake has been found in wheat under ${\text{NH}}_{4}^{+}$ nutrition (e.g., Gashaw and Mugwira, 1981; Wang and Below, 1998), although results varied among cultivars (Gashaw and Mugwira, 1981). For example, ${\text{NH}}_{4}^{+}$ nutrition decreased whole plant and shoot accumulations of K, Cu, Ca, Mg, Fe, Mn, and Zn (Wang and Below, 1998). Nutrient allocation to plant tissues also varied between N forms. ${\text{NH}}_{4}^{+}$-fed plants distributed a smaller percentage of total P, K, Cu, and B to roots relative to ${\text{NO}}_{3}^{+}$-fed plants (Wang and Below, 1995, 1998). Also, a greater percentage of reduced N was allocated to the shoots in ${\text{NH}}_{4}^{+}$-fed plants (Wang and Below, 1995).

Elevated atmospheric concentrations of CO_2_ alter growth and N dynamics of wheat and other C_3_ plants. Under elevated CO_2_, wheat has lower protein and N concentrations (e.g., Thompson and Woodward, 1994; Bloom et al., 2002; Wu et al., 2004), and lower macro- and micronutrients concentrations (Manderscheid et al., 1995; Fangmeier et al., 1997, 1999; Wu et al., 2004; Högy and Fangmeier, 2008). Grain phytate concentrations are also thought to increase or remain the same under elevated CO_2_, and in conjunction with decreased concentrations of micronutrients, bioavailable Zn and Fe are expected to decrease even further under elevated CO_2_ (Loladze, 2002; Manoj-Kumar, 2011), as these micronutrients form indigestible complexes with phytate. By contrast, wheat yields (Fangmeier et al., 1996; Amthor, 2001; Högy and Fangmeier, 2008), harvest index (HI; Wu et al., 2004), whole plant biomass (Fangmeier et al., 1996; Högy and Fangmeier, 2008), shoot biomass (Fangmeier et al., 1996; Högy et al., 2009), and root biomass (Chaudhuri et al., 1990; Wechsung et al., 1995) typically increase under CO_2_ enrichment. In addition, elevated CO_2_ concentration can increase tillering (Weigel et al., 1994), nitrogen use efficiency (NUE, Fangmeier et al., 1997), and micro/macronutrient use efficiencies (Manderscheid et al., 1995). The influence of elevated CO_2_ on many of these characteristics may vary among cultivars and research protocols (e.g., FACE vs. controlled environment chamber, greenhouse vs. field; Amthor, 2001; Högy and Fangmeier, 2008).

Wheat grown under CO_2_ enrichment behaves differently under ${\text{NO}}_{\text{3}}^{-}$ and ${\text{NH}}_{4}^{+}$ nutrition. Exposure to elevated CO_2_ inhibits ${\text{NO}}_{\text{3}}^{-}$ photoassimilation in wheat (Bloom et al., 1989, 2002, 2010; Cousins and Bloom, 2004) as well as in all other C_3_ and C_3_–C_4_ intermediate plants tested (Bloom et al., 2012). At elevated CO_2_, ${\text{NH}}_{4}^{+}$-fed plants showed greater increases in leaf area and smaller decreases in shoot protein concentration than ${\text{NO}}_{\text{3}}^{-}$-fed plants (Bloom et al., 2002), which could have consequences for human nutrition. Vegetative plants receiving ${\text{NH}}_{4}^{+}$ had greater shoot, stem, and root biomass at elevated CO_2_ (Bloom et al., 2002). Wheat receiving ${\text{NO}}_{\text{3}}^{-}$ grew slower at elevated CO_2_ than at ambient CO_2_ (Bloom et al., 2002). Shoot ${\text{NO}}_{\text{3}}^{-}$ concentrations in ${\text{NH}}_{4}^{+}$-fed plants were undetectable while those in ${\text{NO}}_{\text{3}}^{-}$-fed plants increased by 62% with CO_2_ enrichment (Bloom et al., 2002). This increase was associated with an inhibition in ${\text{NO}}_{\text{3}}^{-}$ and ${\text{NO}}_{\text{2}}^{-}$ reductase activities under elevated CO_2_ (Bloom et al., 2002).

The interaction between atmospheric CO_2_ concentration and inorganic N form and how it influences plant growth and nutrient concentrations has not been examined in wheat or any other crop species grown to senescence. Here, we grew wheat hydroponically in controlled environment chambers and measured mineral nutrition, biomass, and nutrient allocation in response to three concentrations of atmospheric CO_2_ (subambient, ambient, and elevated) and two forms of N nutrition (${\text{NH}}_{4}^{+}$ and ${\text{NO}}_{\text{3}}^{-}$). We tested the following hypotheses: (1) plant nutrient concentrations and allocation patterns will respond differently to CO_2_ enrichment under the two N forms, and (2) ${\text{NO}}_{\text{3}}^{-}$-fed plants will show a smaller biomass and yield enhancement in response to CO_2_ enrichment than ${\text{NH}}_{4}^{+}$-fed plants as a result of CO_2_ inhibition of shoot ${\text{NO}}_{\text{3}}^{-}$ assimilation. Also, we observed both differences in the Zn concentration between plants grown on ${\text{NH}}_{4}^{+}$ and ${\text{NO}}_{\text{3}}^{-}$ and a strong dependence of Zn absorption on Zn and phytate concentration, indicating that phytate and bioavailable Zn are affected by N form and CO_2_. Therefore, we used the well supported Miller equation (Miller et al., 2007) to estimate how N and CO_2_ might impact a hypothetical human population. Iron, another important micronutrient that forms complexes with phytate, was not analyzed because we observed no significant differences in iron concentrations between the N forms and because how best to estimate Fe absorption in humans is still uncertain (Welch and Graham, 2004).

## Materials and Methods

### Experimental

Wheat seeds (*Triticum aestivum* cv. Veery 10) were surface sterilized for one minute in 2.6% sodium hypochlorite solution and thoroughly rinsed with DDI water. The seeds were then rolled up in germination paper saturated with 10 mM CaSO_4_. The germination paper was placed in a 400 mL beaker with approximately 75 mL of 10 mM CaSO_4_ solution, covered with a plastic bag and placed in an incubator (23°C) for four days. Seedlings were transplanted into 20 L tubs filled with an aerated nutrient solution that contained 1 mM CaSO_4_, 1 mM K_2_HPO_4_, 1 mM KH_2_PO_4_, 2 mM MgSO_4_, and 0.2 g L^−1^ Fe-NaEDTA and micronutrients (20% of a modified Hoagland’s solution with either 0.2 mM KNO_3_ or 0.1 mM (NH_4_)_2_HPO_4_ as the N source, Epstein and Bloom, 2005). The nutrient solution was replaced weekly and an additional 0.2 mM of ${\text{NO}}_{\text{3}}^{-}$- or ${\text{NH}}_{4}^{+}-N$ was added midweek until harvest. The solution volume was maintained by daily addition of deionized water. Solution pH varied between 6.8 and 7.0 for both of the N forms, and the ${\text{NH}}_{4}^{+}$ and the ${\text{NO}}_{\text{3}}^{-}$ solutions did not differ by more than 0.1 pH units.

The plants were grown in controlled environment chambers (Conviron, Winnipeg, Canada) set at 23/20°C day/night at 60–70% relative humidity with a photoperiod of 15 h. The photosynthetic flux density was 375 μmol m^−2^ s^−1^ at plant height. Plants were subjected to one of three CO_2_ concentrations: “subambient” (310 ± 30 ppm), “ambient” (410 ± 30 ppm), and “elevated” (720 ± 5 ppm). Subambient CO_2_ concentrations were maintained by passing air that entered the growth chamber through wet soda lime, a mixture of KOH, NaOH, and Ca(OH)_2_ that was replaced as needed. The elevated CO_2_ conditions were maintained in an environmental chamber equipped with non-dispersive infrared analyzers for CO_2_ (Horiba model APBA-250E) and valves that added pure CO_2_ to the incoming air stream to hold the chamber concentration at 720 ppm.

The wheat was grown until all aboveground parts turned completely yellow. Plant matter was sorted into grain, chaff, shoots, and roots and dried for 48 h at 55°C. Data on kernel number (KN), kernel mass, number of heads, kernels head^−1^, and HI were collected prior to sample preparation for nutrient analysis. A portion of the grain was analyzed for phytate using a modification of the method as described by Haug and Lantzsch (1983). The remainder of the grain as well as the shoots and chaff was bulked into five repetitions per treatment and sent to the UC Davis Analytical Laboratory for nutrient analysis. The roots of plants for each CO_2_ × N treatment became entangled within the same tub; therefore, we were unable to separate the roots of the individual plants for analysis. Root data are thus presented as means for each treatment with no standard errors or confidence intervals.

Data were analyzed using PROC MIXED (SAS 9.0 Cary, NC, USA). Nitrogen form and CO_2_ factors were treated as fixed independent variables. We used the Tukey–Kramer Honestly Significant Difference test for mean separation. Probabilities less than 0.05 were considered significant. Because some of the transformed variables did not meet the assumption of homogeneity of variances, but one-way ANOVAs met the ANOVA assumptions, we analyzed the results via one-way ANOVAs to gain some information on the interactions between CO_2_ and N form.

### Modeling the influence of N form on Zn nutrition in the human diet

We used a database derived from the United Nation’s Food and Agriculture Organization (FAO)’s national food balance sheets (FBS) to estimate the average daily per capita dietary intake of zinc and phytate from 95 different food commodities in each of 176 countries. This database combines FAO data on per capita intake of food commodities with USDA data on the nutrient or phytate content of each of these commodities. More detailed discussion of the creation of this database for the International Zinc Collaborative Group may be found in Wuehler et al. (2005). Using this database, we produced two datasheets: one containing per capita daily dietary intake of zinc from each food commodity for each country and another containing per capita phytate intake from each food commodity for each country. To calculate total dietary zinc (TDZ) and total dietary phytate (TDP) per country, we summed across the rows of all food commodities for each respective country.

To determine the proportion of a population at risk for zinc deficiency from a hypothetical least developed country (LDC), we first calculated TDP and TDZ values for a set of 44 countries defined by the United Nations as being least developed. We took the mean TDP and TDZ values for these countries to represent a hypothetical “less developed country.” To calculate the bioavailable zinc portion (TAZ; usually a small fraction of TDZ) we used the Miller equation (Equation 1: Miller et al., 2007).

$$\begin{array}{l} \text{TAZ}=0.5\cdot \left(A_{\max}+\text{TDZ}+K_{\text{R}}\cdot \left(1+\frac{\text{TDP}}{K_{\text{P}}}\right)\right.\\ \;\left.-\sqrt{{\left(A_{\max}+\text{TDZ}+K_{\text{R}}\cdot \left(1+\frac{\text{TDP}}{K_{\text{P}}}\right)\right)}^{2}-4\cdot A_{\max}\cdot \text{TDZ}}\right) \end{array}$$

Equation 1: Miller equation

Mean TDZ and TDP values were converted to mg mmol^−1^ and put into the Miller equation to compute the average per capita TAZ in our hypothetical LDC. The variables TDZ, TDP, and TAZ are described above, and *A*_max_, *K*_P_, and *K*_R_ are constants as described in Miller et al. (2007).

We made an assumption that our hypothetical LDC receives half of its phytate and half of its zinc from wheat, which is roughly consistent with many of the LDCs in the FAO database. We analyzed the effect of elevated carbon dioxide levels on TDP, TDZ, and TAZ concentrations in a hypothetical LDC population for both ${\text{NH}}_{4}^{+}$ and ${\text{NO}}_{\text{3}}^{-}$-supplied wheat. To calculate a new TAZ for wheat grown under elevated CO_2_ conditions, we first calculated the percent change in TAZ from ambient to elevated levels for wheat receiving ${\text{NH}}_{4}^{+}$ or ${\text{NO}}_{\text{3}}^{-}.$ This computed percent change was then applied to half of the hypothetical TDZ and TDP; meanwhile, the other half of the hypothetical TDZ and TDP remained unmodified. Thus, the total new TDP and TDZ is the sum of the unmodified and modified portions. These new TDP and TDZ values for both ${\text{NH}}_{4}^{+}$ and ${\text{NO}}_{\text{3}}^{-}$-supplied wheat were then put into the Miller equation to compute new hypothetical TAZ values for an LDC. Differences and corresponding percent changes between the new TAZ values and the original TAZ value for a LDC were computed to determine the overall affect of elevated CO_2_ on TAZ in ${\text{NH}}_{4}^{+}$ and ${\text{NO}}_{\text{3}}^{-}$-supplied wheat for an average developing world population. TAZ, TDP, and TDZ concentrations can only be compared within a single N form across the CO_2_ concentrations due to methodological constraints of the model.

## Results

We divide the results here into three categories: first, biomass and yield data for the shoots, grain, and roots; second, tissue concentrations and whole plant micro- and macronutrient contents; and third, nutrient distribution among the different tissues. Values of the statistical significance of the results were place into a table (Table 1) in order to improve the readability of the text.

**Table 1:** **Results of a series of one-way ANOVAs run on the data**.

Among CO_2_ cnc. within an N form	Grain		Shoot			
	${\text{NH}}_{4}^{+}$	${\text{NO}}_{\text{3}}^{-}$	${\text{NH}}_{4}^{+}$	${\text{NO}}_{\text{3}}^{-}$		
Total N	**	NS	***	NS		
P	*	NS	**	NS		
K	***	NS	NS	***		
S	NS	NS	**	NS		
Ca	***	NS	*	**		
Mg	NS	NS	NS	***		
Zn	NS	NS	***	***		
B	**	*	*	*		
Mn	**	NS	***	NS		
Fe	*	NS	*	*		
Cu	***	NS	NS	***		
${\text{NO}}_{\text{3}}^{-}\;-\text{N}$	*	NS	***	***		
Phytate	NS	NS	N/A	N/A		

**Between N forms within a CO_2_ cnc**.	**Grain**			**Shoot**		
	**Sub**	**Amb**	**Elev**	**Sub**	**Amb**	**Elev**

Total N	**	NS	NS	NS	NS	**
P	NS	NS	NS	*	NS	**
K	**	NS	**	NS	**	NS
S	*	NS	*	**	NS	***
Ca	***	***	***	NS	NS	NS
Mg	NS	NS	NS	***	***	*
Zn	*	***	*	**	***	***
B	NS	NS	*	***	NS	***
Mn	NS	NS	NS	***	***	*
Fe	NS	NS	NS	NS	NS	NS
Cu	**	**	**	NS	***	*
${\text{NO}}_{\text{3}}^{-}-\text{N}$	NS	NS	NS	***	***	***
Phytate	**	NS	NS	N/A	N/A	N/A

**Among CO_2_ cnc. or between N forms**	**Sub**	**Amb**	**Elev**	**${\text{NH}}_{4}^{+}$**	**${\text{NO}}_{\text{3}}^{-}$**

Yield	NS	*	NS	**	NS	
Shoot	**	NS	NS	**	NS	
Chaff	NS	*	NS	**	NS	
Grain number	NS	NS	NS	*	NS	
Grain mass	***	NS	NS	***	NS	
Grains head^−1^	***	NS	NS	NS	NS	
Heads	**	**	NS	*	NS	
Harvest index	NS	NS	NS	NS	NS	

Differences among CO_2_ concentrations within an N form and between N forms at each CO_2_ concentration for shoot and grain nutrient concentrations. Differences among CO_2_ concentrations within an N form or between N forms at each CO_2_ concentration for biomass and yield components. The symbols indicate statistical significance (*0.05, **0.01, ***0.001, NS, not significant).

### Biomass and yield

**Figure 1:**
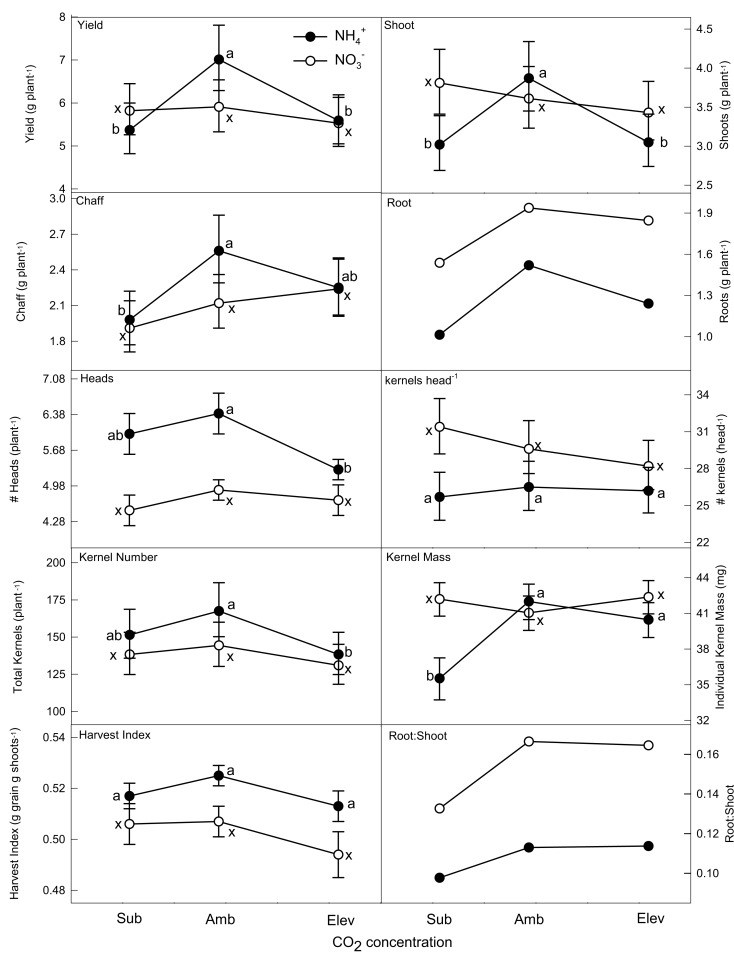
**The effect of N form and CO_2_ concentration on biomass and yield components of wheat hydroponically grown to senescence**. Closed (${\text{NH}}_{4}^{+}$) and open (${\text{NO}}_{\text{3}}^{-}$) symbols and error bars represent the back-transformed means and 95% confidence intervals (*n* = 10). Means within N form across CO_2_ treatment are significantly different if labeled with different letters. Root-related data do not have error bars.

Plants supplied ${\text{NH}}_{4}^{+}\;\text{vs}\text{.}\;{\text{NO}}_{\text{3}}^{-}$ nutrition reacted differently to CO_2_ enrichment (Figure 1; Table 1). Plants supplied ${\text{NH}}_{4}^{+}$ differed across CO_2_ treatments for most of the yield and biomass measurements. The greatest values typically were found at ambient CO_2_ concentrations. Shoot, chaff, grain yield, number of heads, and KN were greatest at ambient CO_2_ levels. Individual kernel mass was greatest under both ambient and elevated CO_2_ treatments. HI and kernels head^−1^ showed no change across CO_2_ treatments. In contrast, biomass and yield measures of ${\text{NO}}_{\text{3}}^{-}$-supplied plants did not differ among the three CO_2_ concentrations.

At subambient CO_2_, differences between the ${\text{NH}}_{4}^{+}$ and ${\text{NO}}_{\text{3}}^{-}$ treatments occurred in shoot biomass and three of the yield components: kernel mass, head number, and kernels head^−1^. Ammonium-supplied plants had a larger number of heads while ${\text{NO}}_{\text{3}}^{-}$-supplied plants had greater shoot biomass, kernel mass, and kernels head^−1^. At ambient CO_2_, ${\text{NH}}_{4}^{+}$-supplied plants had a greater number of heads and greater chaff biomass. Plants supplied ${\text{NO}}_{\text{3}}^{-}$ had a larger number of kernels head^−1^. At elevated CO_2_, biomass and yield measures did not differ with N treatment.

### Root

Roots had a smaller mean biomass when supplied ${\text{NH}}_{4}^{+}$ than when supplied ${\text{NO}}_{\text{3}}^{-}$ at all CO_2_ concentrations (Figure 1). Both N treatments had the greatest biomass at ambient CO_2_, and the smallest at subambient CO_2_. The highest root:shoot ratios for both ${\text{NH}}_{4}^{+}$ and ${\text{NO}}_{\text{3}}^{-}$-supplied plants were observed at ambient and elevated CO_2_. Ammonium-supplied plants always had lower root:shoot ratios and biomasses than ${\text{NO}}_{\text{3}}^{-}$-supplied plants at the same CO_2_ concentration.

### Nutrients

#### Total plant nutrients

Total plant nutrients generally followed the same trend within N form, although ${\text{NH}}_{4}^{+}$-supplied plants exhibited a greater diversity of responses to increasing CO_2_ concentrations (Table 2). Total plant P, K, B, Ca, Mg, and Zn decreased with increasing CO_2_ under ${\text{NH}}_{4}^{+},$ while S and Mn were highest under ambient CO_2_. Ammonium-supplied plants had the greatest amounts of Fe and total N at subambient CO_2_. Nitrate-supplied plants accumulated the greatest amounts of total N, P, K, S, B, Ca, Zn, Mn, and Mg at ambient CO_2_. Only three nutrients – K, S, and Fe – had the lowest contents at elevated CO_2_.

**Table 2:** **Total plant nutrients (mg plant^−^^1^) as affected by N form and CO_2_ concentration**.

	Sub	Amb	Elev	Sub	Amb	Elev	Sub	Amb	Elev	Sub	Amb	Elev
		**Total N**			**P**			**K**			**S**	
${\text{NH}}_{4}^{+}$	215.66	191.62	208.56	80.64	73.96	68.69	228.91	202.92	196.33	49.98	50.72	46.82
${\text{NO}}_{\text{3}}^{-}$	159.39	210.26	164.88	63.21	85.02	67.75	208.32	259.07	198.79	42.21	50.84	38.25
		**B**			**Ca**			**Mg**			**Zn**	
${\text{NH}}_{4}^{+}$	0.28	0.25	0.18	23.18	19.55	19.24	42.41	38.62	35.34	0.62	0.54	0.45
${\text{NO}}_{\text{3}}^{-}$	0.29	0.41	0.31	21.10	25.54	22.48	45.26	52.45	52.45	0.27	0.48	0.36
		**Mn**			**Fe**			**Cu**				
${\text{NH}}_{4}^{+}$	2.66	2.93	2.24	1.93	1.26	1.47	0.06	0.05	0.05			
${\text{NO}}_{\text{3}}^{-}$	2.16	3.54	2.52	2.16	2.71	1.75	0.05	0.06	0.06			

#### Shoot

**Figure 2:**
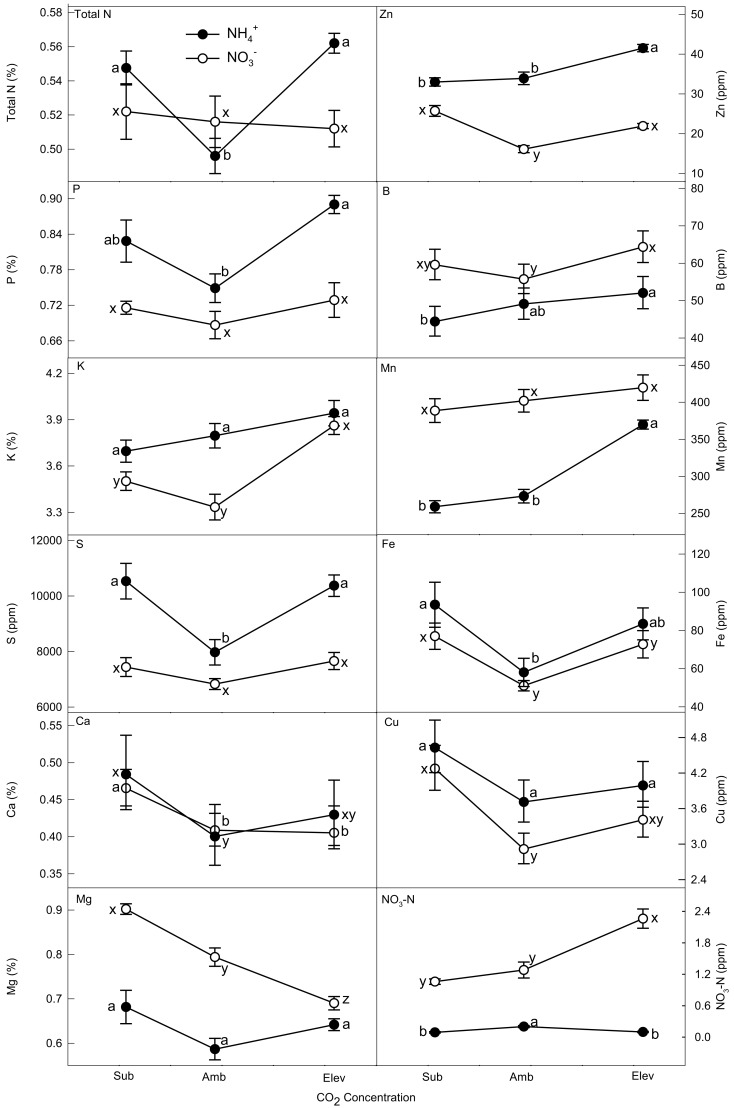
**The effect of N form and CO_2_ concentration on shoot nutrient concentrations of wheat grown hydroponically to senescence**. Closed (${\text{NH}}_{4}^{+}$) and open (${\text{NO}}_{\text{3}}^{-}$) symbols represent back-transformed means and 95% confidence intervals (*n* = 5). Macro- and micronutrients are listed in the upper left of each frame. Differences are significant within N form if letters are different. Differences between N forms at each CO_2_ concentration are generally significantly different if error bars do not overlap (see Table 1 for statistical significance).

Under ${\text{NH}}_{4}^{+}$ supply, plants varied with CO_2_ concentration for total N, P, S, Ca, Cu, B, Mn, Zn, and ${\text{NO}}_{\text{3}}^{-}$ (Table 1; Figure 2). Calcium and Cu were highest under subambient CO_2_. Total N and S were greatest at subambient and elevated CO_2_. Nitrate-N was greatest at ambient CO_2_. Phosphorus was highest at elevated CO_2_ concentrations. Boron, Zn, and Mn increased with CO_2_ concentration.

Plants supplied ${\text{NO}}_{\text{3}}^{-}$ showed significant variation across CO_2_ treatments for K, Ca, Mg, B, Fe, Cu, Zn, and ${\text{NO}}_{\text{3}}^{-}-N$ (Table 1; Figure 2). Calcium and Cu had the greatest concentrations at subambient CO_2_. The highest concentrations of B, Fe, and Zn occurred at subambient and elevated CO_2_. Potassium concentrations were highest at elevated CO_2_. Nitrate-N increased with CO_2_. Magnesium showed the opposite trend, decreasing with CO_2_ concentration.

Differences between N forms were also evident. At subambient CO_2_, ${\text{NH}}_{4}^{+}$-supplied plants had increased concentrations of P, S, and Zn, while ${\text{NO}}_{\text{3}}^{-}$-supplied plants had greater concentrations of B, Mg, Mn, and ${\text{NO}}_{\text{3}}^{-}-N$ (Table 1; Figure 2). Concentrations of K, Zn, and Cu were higher in plant supplied ${\text{NH}}_{4}^{+}$ at ambient CO_2_, while Mg, Mn, and ${\text{NO}}_{\text{3}}^{-}-N$ were greater in plants supplied ${\text{NO}}_{\text{3}}^{-}.$ At elevated CO_2_, concentrations of N, P, S, and Zn were higher in plants supplied ${\text{NH}}_{4}^{+},$ while concentrations of B, Mg, Mn, and ${\text{NO}}_{\text{3}}^{-}-N$ were greater in plants supplied ${\text{NO}}_{\text{3}}^{-}.$

#### Grain

##### Grain nutrient concentrations

**Figure 3:**
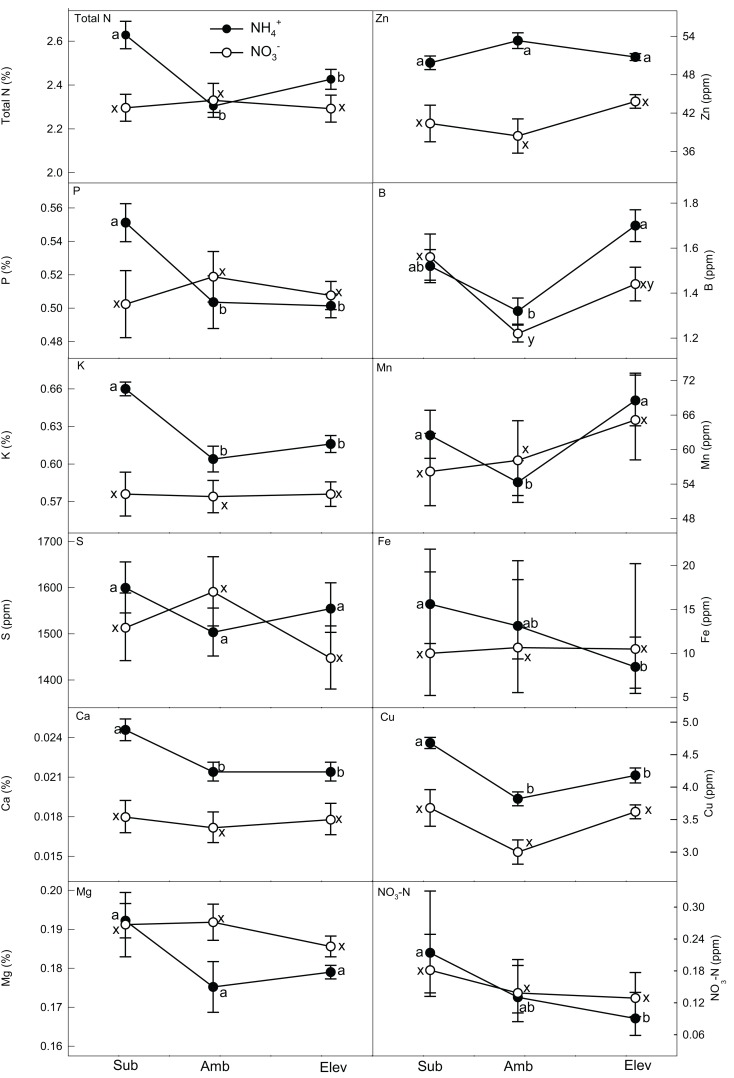
**The effect of N form and CO_2_ concentration on grain nutrient concentrations of wheat grown hydroponically to senescence**. Closed (${\text{NH}}_{4}^{+}$) and open (${\text{NO}}_{\text{3}}^{-}$) symbols represent back-transformed means and 95% confidence intervals (*n* = 5). Macro- and micronutrients are listed in the upper left of each frame. Differences are significant within N form if letters are different. Differences between N forms at each CO_2_ concentration are generally significantly different if error bars do not overlap (see Table 1 for statistical significance).

Plants supplied ${\text{NH}}_{4}^{+}$ showed significant variation across the CO_2_ treatments in the concentrations of total N, P, K, Ca, B, Cu, Fe, Mn, and ${\text{NO}}_{\text{3}}^{-}-N$ (Table 1; Figure 3). The greatest concentrations of total N, P, K, Ca, and Cu were found at subambient CO_2_. Iron concentrations were high at both subambient and ambient CO_2_. Boron was equally high at subambient and elevated CO_2_. Manganese was greatest at elevated CO_2_. Nitrate-N decreased with increasing CO_2_.

Significant differences among the ${\text{NO}}_{\text{3}}^{-}$-supplied plants across CO_2_ treatments were only observed in S and B. The greatest concentrations of B were found at subambient CO_2_. Sulfur was highest at ambient CO_2_.

Nitrogen form significantly affected grain nutrient concentrations (Table 1; Figure 3). At subambient CO_2_, ${\text{NH}}_{4}^{+}$-supplied plants had higher concentrations of total N, K, S, Ca, Zn, and Cu than ${\text{NO}}_{\text{3}}^{-}$ plants. At ambient CO_2_, Ca, Zn, and Cu were greatest under ${\text{NH}}_{4}^{+}.$ Ammonium-supplied plants also had the highest concentrations of K, S, Ca, Zn, and Cu at elevated CO_2_. At no CO_2_ concentration did plants supplied ${\text{NH}}_{4}^{+}$ have significantly lower concentrations of any micro- or macronutrient than those supplied ${\text{NO}}_{\text{3}}^{-}.$

##### Phytate and bioavailable Zn

**Figure 4:**
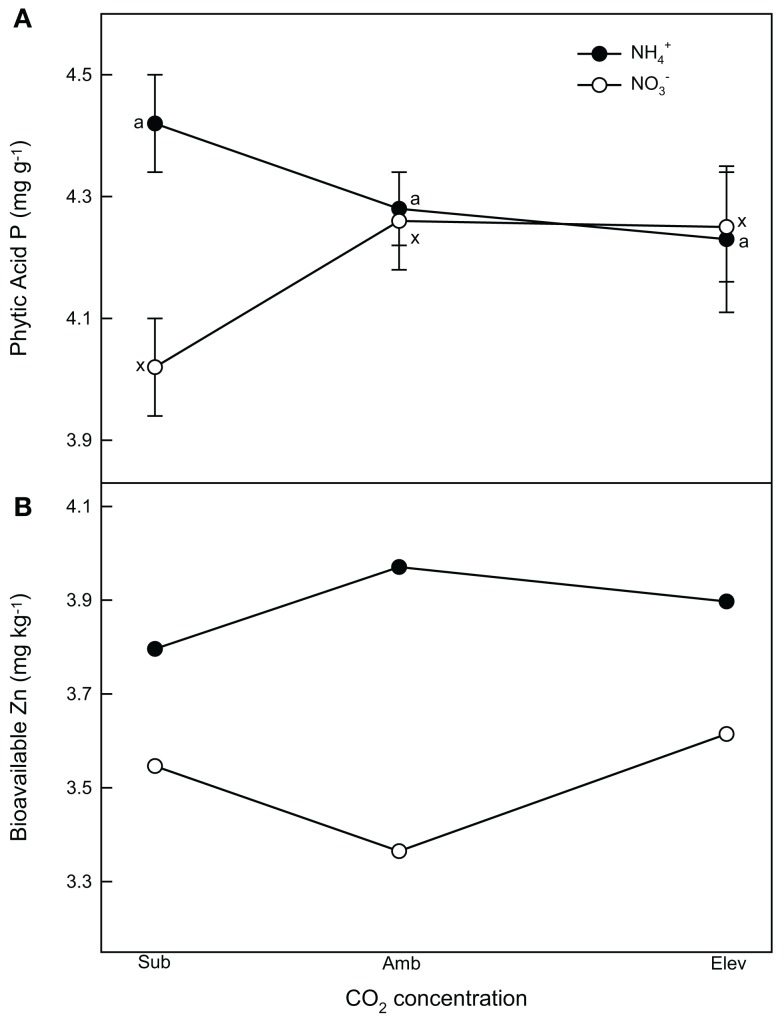
**The effect of N form and CO_2_ concentration on (A) grain phytate (phytic acid) and (B) bioavailable Zn concentrations of wheat grown hydroponically to senescence**. Closed (${\text{NH}}_{4}^{+}$) and open (${\text{NO}}_{\text{3}}^{-}$) symbols represent the back-transformed means and 95% confidence intervals (*n* = 4). The model used to calculate the bioavailable Zn did not allow the inclusion of confidence intervals.

Phytate was relatively insensitive to CO_2_ concentration. Phytate concentrations were highest at subambient CO_2_ for ${\text{NH}}_{4}^{+}$-supplied plants (Figure 4). Subambient CO_2_ also produced the lowest phytate concentrations in ${\text{NO}}_{\text{3}}^{-}$-supplied plants. ${\text{NH}}_{4}^{+}$-supplied plants had greater phytate concentrations than ${\text{NO}}_{\text{3}}^{-}$-supplied plants at subambient CO_2_, but not at the other CO_2_ concentrations. Grain from plants grown under ${\text{NH}}_{4}^{+}$ nutrition had roughly 7, 18, and 8% higher bioavailable Zn than ${\text{NO}}_{\text{3}}^{-}$-supplied plants at subambient, ambient, and elevated CO_2_, respectively (Figure 4).

Based on this phytate and bioavailable Zn data, we modeled how a human population from a LDC would be affected by changes in atmospheric CO_2_ concentrations (Table 3). The calculations were based on differences among CO_2_ concentrations; therefore, modeled TDZ, TDP, and TAZ values cannot be compared between ${\text{NH}}_{4}^{+}$ and ${\text{NO}}_{\text{3}}^{-}$-supplied grain. Grain from plants supplied the different N forms behaved differently as CO_2_ concentration increased. We found that under ${\text{NH}}_{4}^{+}$ supply, TAZ would increase 3.6% with the rise in CO_2_ from subambient to ambient, and decrease 1.6% with the rise from ambient to elevated CO_2_ (Figure 4). Humans provided ${\text{NO}}_{\text{3}}^{-}$-supplied wheat would experience a decrease in TAZ of 3.5% going from subambient to ambient, and an increase 5.6% from ambient to elevated CO_2_ (Figure 4).

**Table 3:** **Total dietary Zn (TDZ), total dietary phytate (TDP), and total bioavailable Zn (TAZ) of a human population from a hypothetic less developed nation reliant on wheat for 50% of their dietary phytate and Zn as modeled using the Miller equation**.

		Sub → Amb (g/kg^−1^)	Amb → Elev (g/kg^−1^)
	TDZ	9.21	8.69
${\text{NH}}_{4}^{+}$	TDP	2241.92	2264.70
	TAZ	1.76	1.67
	TDZ	8.68	9.53
${\text{NO}}_{\text{3}}^{-}$	TDP	2346.00	2275.33
	TAZ	1.64	1.79

*The data columns represent the change in TDZ, TDP, and TAZ concentration going from subambient to ambient and ambient to elevated CO_2_ concentrations, respectively. The values are calculated as deviations from the mean TDZ, TDP, and TAZ concentrations as produced from FAO and USDA data (Wuehler et al., 2005). Baseline values for TDZ, TDP, and TAZ were 8.90, 2278.00, and 1.70 g kg^−1^, respectively*.

#### Roots

**Figure 5:**
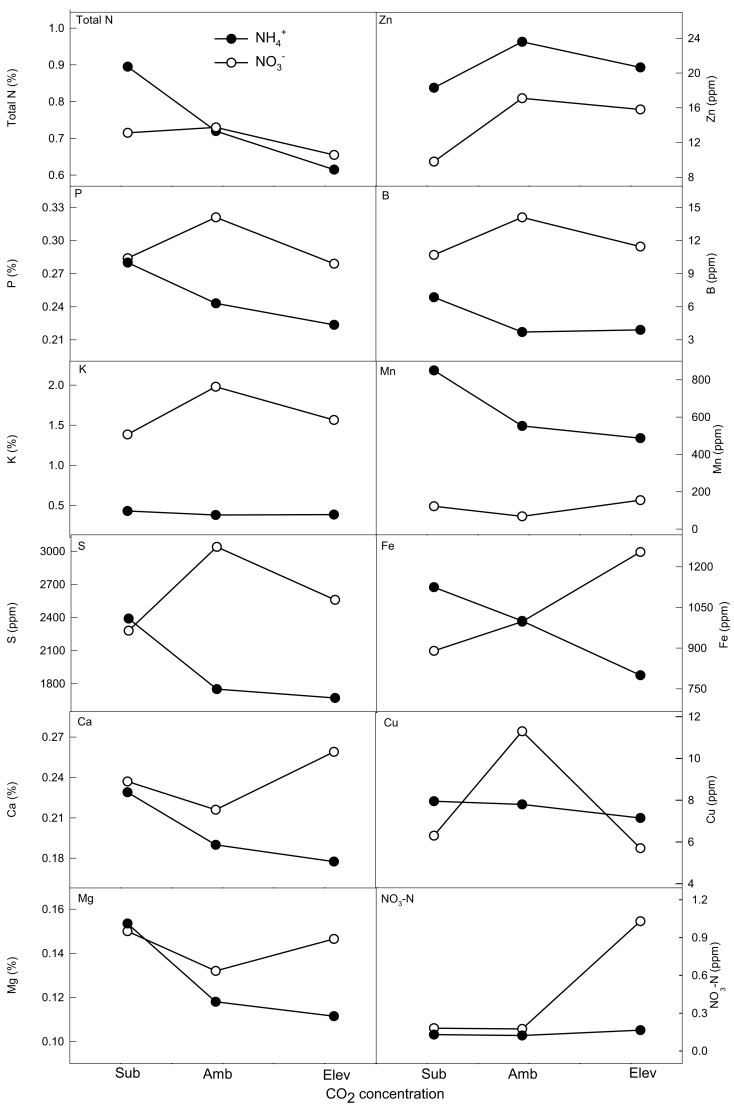
**The effect of N form and CO_2_ concentration on root tissue nutrient concentrations of wheat grown hydroponically to senescence**. Closed (${\text{NH}}_{4}^{+}$) and open (${\text{NO}}_{\text{3}}^{-}$) symbols represent the bulked treatment mean (*n* = 10). Macro- and micronutrients are listed in the upper left of each frame. The lack of error bars reflects that the root mass for each treatment was bulked and analyzed as a unit.

Ammonium-supplied plants generally showed a trend toward decreasing nutrient concentrations with increasing CO_2_ concentration while ${\text{NO}}_{\text{3}}^{-}$-supplied plants varied widely across CO_2_ treatments (Figure 5). The decrease in nutrient concentrations under ${\text{NH}}_{4}^{+}$ supply corresponded to an increase in root mass. Nitrate-supplied plants tended to have their highest nutrient concentrations in the ambient and elevated CO_2_ treatments. Ammonium-supplied plants had higher concentrations of Zn and Mn across all of the CO_2_ treatments, as well as higher total N and Fe at subambient CO_2_. Nitrate-supplied plants typically had higher concentrations of the other nutrients at all CO_2_ concentrations.

#### Distribution of nutrients

The distribution of nutrients and micronutrients among plant parts followed similar patterns in both the ${\text{NH}}_{4}^{+}$ and ${\text{NO}}_{\text{3}}^{-}$-supplied plants, although the ${\text{NH}}_{4}^{+}$-supplied plant distributions were slightly more variable (Table 4). Allocations to root and grain usually were greatest at ambient CO_2_, and those to chaff and shoots at either subambient or elevated CO_2_. Grain typically contained the largest proportion of total N, P, Zn, and Cu, although the organ with the largest percentage of Cu varied with CO_2_ treatment among ${\text{NO}}_{\text{3}}^{-}$-supplied plants. Plants at subambient and elevated CO_2_ allocated more Cu to the grain, while those at ambient CO_2_ allocated more to the roots. In general shoots received the majority of K, S, B, Ca, and Mg for all N and CO_2_ treatments. Ammonium-supplied plants allocated slightly more Mn to the roots at subambient CO_2_, but allocated increasing amounts to the shoots at the expense of the roots as CO_2_ concentration increased. In contrast, ${\text{NO}}_{\text{3}}^{-}$-supplied plants allocated most of the Mn to the shoots. Ammonium-supplied plants typically allocated more resources to the chaff while ${\text{NO}}_{\text{3}}^{-}$-supplied plants allocated a greater percentage of elements to the roots.

**Table 4:** **Organ nutrient allocation as percentage of the plant total under the CO_2_ and N form treatments**.

		Root	Cha	Shoots	Grain	Root	Chaff	Shoots	Grain	Root	Chaff	Shoots	Grain	Root	Chaff	Shoots	Grain
		**Total N**				**P**				**K**				**S**			
**Sub**	${\text{NH}}_{4}^{+}$	5.22	9.23	10.35	75.20	4.34	14.68	38.99	41.98	2.24	22.75	58.15	16.86	4.71	16.67	63.11	15.50
**Amb**		5.02	6.36	9.08	79.54	4.56	12.10	36.78	46.55	2.38	16.30	62.59	18.73	5.18	11.25	61.49	22.09
**Elev**		4.32	8.36	9.79	77.52	4.13	12.96	40.89	42.02	2.48	16.33	63.12	18.06	4.12	14.88	63.54	17.46
**Sub**	${\text{NO}}_{\text{3}}^{-}$	6.45	4.66	11.89	77.00	6.46	10.21	41.24	42.10	10.05	10.50	64.04	15.41	8.14	5.21	66.52	20.12
**Amb**		7.96	4.95	10.48	76.61	9.03	10.91	35.97	44.09	17.87	10.54	56.04	15.56	13.60	7.87	56.96	21.57
**Elev**		6.67	6.88	9.82	76.64	7.30	12.78	36.04	43.88	12.74	12.44	59.35	15.48	10.93	7.19	61.52	20.35
		**B**				**Ca**				**Mg**				**Zn**			
**Sub**	${\text{NH}}_{4}^{+}$	3.53	23.50	69.11	3.86	10.74	14.26	69.31	5.69	4.25	12.40	57.24	26.11	4.57	9.23	24.98	61.22
**Amb**		2.03	23.61	70.72	3.63	12.45	11.71	68.88	6.95	4.13	12.12	53.64	30.11	5.57	11.31	20.97	62.15
**Elev**		2.07	25.21	68.62	4.10	11.44	13.49	68.80	6.27	3.80	14.35	54.20	27.66	5.12	12.16	25.49	57.23
**Sub**	${\text{NO}}_{\text{3}}^{-}$	5.25	17.24	74.74	2.77	14.82	7.12	73.89	4.17	4.34	9.17	66.09	20.40	4.17	5.75	27.20	62.88
**Amb**		9.11	21.42	67.10	2.37	18.83	10.06	66.59	4.53	5.19	13.73	58.29	22.78	9.56	8.51	16.76	65.16
**Elev**		6.38	23.09	67.86	2.67	21.56	9.90	63.65	4.89	6.12	13.90	54.40	25.58	7.14	8.96	18.65	65.25
		**Mn**				**Fe**				**Cu**							
**Sub**	${\text{NH}}_{4}^{+}$	38.53	11.97	35.41	14.09	72.81	3.39	18.44	5.36	15.77	10.71	28.01	45.52				
**Amb**		30.72	14.50	39.80	14.98	81.28	1.23	12.21	5.29	19.64	8.48	24.49	47.39				
**Elev**		23.01	18.98	43.24	14.78	74.85	2.01	19.47	3.67	18.00	9.19	24.97	47.84				
**Sub**	${\text{NO}}_{\text{3}}^{-}$	7.72	16.35	62.77	13.16	78.58	0.92	16.79	3.70	18.96	8.27	32.51	40.26				
**Amb**		5.47	20.25	60.01	14.26	87.37	0.74	8.39	3.50	40.79	6.79	19.64	32.78				
**Elev**		10.15	23.71	52.07	14.07	87.06	0.70	9.46	2.78	21.70	8.50	24.48	45.32				

## Discussion

No other study to our knowledge has examined the influence of N form (${\text{NH}}_{4}^{+}$ vs. ${\text{NO}}_{\text{3}}^{-}$) on plant nutrient relations at three different atmospheric CO_2_ concentrations. Overall, N form affected growth, total plant nutrient contents, and nutrient distribution in senescing wheat shoots, grain, and roots. The influence of ${\text{NH}}_{4}^{+}$ and ${\text{NO}}_{\text{3}}^{-}$ on growth and nutrient status were so distinct that they should be treated as separate nutrients and not bundled into a general category of N nutrition. Wheat size and nutrition at senescence responded to CO_2_ concentration in a non-linear manner. As was previously shown (Bloom et al., 2012), we found that plants supplied with ${\text{NH}}_{4}^{+}$ were more responsive to CO_2_ concentration than those supplied with ${\text{NO}}_{\text{3}}^{-}.$

Although not explicitly addressed here because of the heterogeneity of variances, interactions between CO_2_ and N treatments likely existed for a number of the biomass and nutrient measures. Most nutrient concentrations were generally higher in ${\text{NH}}_{4}^{+}$-supplied plants, with the exceptions of ${\text{NO}}_{\text{3}}^{-}-N,$ Mg, B, and Mn, which were generally higher in ${\text{NO}}_{\text{3}}^{-}$-supplied plants. Phytate, which hinders human absorption of Zn and Fe (Raboy, 2009), showed little variation at ambient and elevated CO_2_ between ${\text{NH}}_{4}^{+}$ and ${\text{NO}}_{\text{3}}^{-}$-supplied plants, which, in conjunction with the observed greater bioavailable of Zn in ${\text{NH}}_{4}^{+}$-supplied plants, may have consequences for human nutrition. Distribution of nutrients to the shoots, roots, chaff, and grain in response to CO_2_ concentration and N form was also non-linear and varied by nutrient.

### Biomass and yield

The data support our hypothesis that ${\text{NO}}_{\text{3}}^{-}$-supplied plants would show a more limited biomass and yield enhancement with CO_2_ enrichment than ${\text{NH}}_{4}^{+}$-supplied plants. Nevertheless, mean biomass and yield decreased from ambient to elevated CO_2_ in both ${\text{NO}}_{\text{3}}^{-}$- and ${\text{NH}}_{4}^{+}$-supplied plants in contrast to biomass increases in prior work on wheat seedlings (Bloom et al., 2002). ${\text{NO}}_{\text{3}}^{-}$-supplied plants allocated more biomass to roots and had larger root:shoot ratios than ${\text{NH}}_{4}^{+}$-supplied plants regardless of CO_2_ concentrations as has been reported previously (Wang and Below, 1995; Bloom et al., 2002), but increased root mass at elevated CO_2_ concentration for ${\text{NO}}_{\text{3}}^{-}$-supplied plants reported previously (Bloom et al., 2002) were not observed here. The shoot biomass data suggest that growth differences measured early in the lifespan of wheat supplied with ${\text{NH}}_{4}^{+}$ or ${\text{NO}}_{\text{3}}^{-}$ or ${\text{NH}}_{4}^{+}$ (i.e., greater shoot biomass in plants supplied ${\text{NH}}_{4}^{+}$ relative to those supplied ${\text{NO}}_{\text{3}}^{-}$ at elevated CO_2_ concentrations; Bloom et al., 2002) do not necessarily carry through to senescence. This may be due in part to a shift in ${\text{NO}}_{\text{3}}^{-}$ assimilation to the root (Kruse et al., 2003), allowing ${\text{NO}}_{\text{3}}^{-}$-supplied plants to compensate for the decrease in shoot ${\text{NO}}_{\text{3}}^{-}$ assimilation that occurs at elevated atmospheric CO_2_ concentrations (Bloom et al., 2002, 2010, 2012).

The decrease in yield and biomass measures at elevated CO_2_ concentrations does not agree with field observations where wheat yields as well as overall biomass increased with elevated CO_2_ (Högy and Fangmeier, 2008; Taub et al., 2008). Similarly, our results that the greatest values for other yield measures (e.g., heads, kernel mass, KN) occurred at ambient CO_2_ concentrations varies from the literature. High CO_2_ has been found to increase flowering tillers (Havelka et al., 1984; Fangmeier et al., 1996), KN (McKee et al., 1997), and kernel mass (i.e., thousand grain weight; McKee et al., 1997). Conflicting results, however, have also been reported (e.g., Havelka et al., 1984). Many of the field and open top chamber studies were grown under natural light and thus received substantially greater photosynthetic flux density than our chamber-grown plants. These higher light conditions would be more favorable to biomass accumulation. Also, these studies typically applied high amounts of mixed N fertilizer (e.g., NH_4_NO_3_), and yields and biomass have been found to be greater under mixed N nutrition than under either ${\text{NH}}_{4}^{+}$ or ${\text{NO}}_{\text{3}}^{-}$ alone (Cox and Reisenauer, 1973; Gentry et al., 1989; Heberer and Below, 1989; Wang and Below, 1995). Finally, the wheat cultivar we used (*T. aestivum* cv. Veery 10) is a short-statured variety that has rarely been used in other studies and may have accounted for some of the differences between our study and other published data.

Our results that ${\text{NH}}_{4}^{+}$-supplied plants had greater yield and yield components than ${\text{NO}}_{\text{3}}^{-}$-supplied plants at ambient CO_2_ have been observed previously (Wang and Below, 1996; Chen et al., 1998). Wang and Below (1995) observed greater numbers of kernels head^−1^ and KN in plants supplied ${\text{NO}}_{\text{3}}^{-}$ that was not observed here. Their study, however, supplied ${\text{NH}}_{4}^{+}$ at relatively high levels (~8.9 vs. 0.2 mM ${\text{NH}}_{4}^{+}$ in our study). Several studies (Bennett and Adams, 1970; Cox and Reisenauer, 1973) have found that incipient ${\text{NH}}_{4}^{+}$ toxicity can start appearing at N levels as low as 0.08–0.2 mM ${\text{NH}}_{4}^{+}$, although the onset of ${\text{NH}}_{4}^{+}$ toxicity depends on light level (Magalhaes and Wilcox, 1984; Britto and Kronzucker, 2002) and solution pH (Findenegg, 1987). The poorer performance of the ${\text{NH}}_{4}^{+}$ treatment in Wang and Below (1995), therefore, might derive from ${\text{NH}}_{4}^{+}$ toxicity. We have previously determined that the 0.2 mM ${\text{NH}}_{4}^{+}$-supplied to our plants to be sufficiently high for normal growth, but low enough to avoid toxicity problems under our experimental conditions (Bloom et al., 2002).

### Plant nutrients

Our second hypothesis, that nutrient concentrations are differentially affected by the inorganic N form supplied to the plants and CO_2_ enrichment, was supported by our data. CO_2_ concentration and N form interactions may alter tissue demands for nutrients. For many nutrients, ratios between different elements are typically maintained within a narrow range (Garten, 1976; Bloom et al., 1985; Loladze, 2002). CO_2_ concentration and N form may disturb the balance between different nutrients, leading to a cascade of changes in demand, accumulation, and allocation among the different plant tissues (e.g., Loladze, 2002; Högy and Fangmeier, 2008; Natali et al., 2009). Nitrate-supplied plants accumulated the greatest amounts of nutrients at ambient CO_2_ (Table 2). Some portion of the greater response of ${\text{NH}}_{4}^{+}$-supplied plants to CO_2_ derived from a dilution effect from the greater biomass at ambient CO_2_ concentrations (Figures 2 and 3). Total amounts of nutrients tended to decline with CO_2_ enrichment for ${\text{NH}}_{4}^{+}$-supplied plants, which had the greatest amounts of macro/micronutrients at subambient CO_2_ (Table 2). These results have not been observed in other published studies (e.g., Fangmeier et al., 1997; Wu et al., 2004). Growth chamber studies, however, tend to have more exaggerated differences among treatments than field and greenhouse experiments (Högy and Fangmeier, 2008), and N source cannot be well-controlled in field and greenhouse experiments.

The observed increase in ${\mathrm{NO}}_{3}^{-}-N$ concentration with CO_2_ concentration in ${\text{NO}}_{\text{3}}^{-}$-supplied plants has been reported previously (Bloom et al., 2002), and adds further support to the hypothesis that elevated CO_2_ concentrations and the resulting decrease in photorespiration inhibit shoot ${\text{NO}}_{\text{3}}^{-}$ photoassimilation. Nevertheless, tissue ${\text{NO}}_{\text{3}}^{-}-N$ concentrations observed here were substantially lower than those in the earlier study (Bloom et al., 2002). Again, this may derive from difference in life stages in the two studies. Most of the N available to the plant for grain filling comes from N translocation rather than uptake from the substrate (Simpson et al., 1983). Probably, the plants continued to assimilate plant ${\text{NO}}_{\text{3}}^{-}$ using a non-photorespiratory dependent process such as root assimilation after root N uptake slowed or stopped. Loss of ${\text{NO}}_{\text{3}}^{-}$ through root efflux to the nutrient solution also may have contributed to the lower concentration of ${\text{NO}}_{\text{3}}^{-}-N.$

The partitioning and accumulation of all mineral elements was affected in some manner by the CO_2_ treatment and N form supplied to the plants. Observations that cation concentrations decrease under ${\text{NH}}_{4}^{+}$ supply (e.g., Cox and Reisenauer, 1973; Gashaw and Mugwira, 1981; Wang and Below, 1998) relative to ${\text{NO}}_{\text{3}}^{-}$ supply were not apparent in this study. Again, this could be partly due to the relatively low concentration of ${\text{NH}}_{4}^{+}$-supplied in our study, the age of the plants at harvest, and differences among wheat cultivars.

Allocation of nutrients within the plant followed similar trends for both N forms, with the exceptions of Mn and Cu (Table 2). Interestingly, in ${\text{NO}}_{\text{3}}^{-}$-supplied plants, shoot Mn concentrations increased slightly with CO_2_, and these plants allocated far more Mn to the shoots than ${\text{NH}}_{4}^{+}$-supplied plants at all CO_2_ concentrations. Manganese (Mn^2+^) has been found to activate Rubisco in place of Mg^2+^ and the Rubisco-Mn complex has been observed to decrease Rubisco carboxylase activity while minimally affecting or even enhancing oxygenase activity (Jordan and Ogren, 1983). The slight increase in shoot Mn with CO_2_ corresponded to a large 23% decrease in Mg concentration. Manganese, which can act as a cofactor for glutamine synthetase (Smirnoff and Stewart, 1987), was also the only nutrient that ${\text{NH}}_{4}^{+}$-supplied plants allocated a greater percentage to the roots at the expense of the shoots. ${\text{NO}}_{\text{3}}^{-}$-supplied plants typically allocated a higher percentage of most nutrients to the roots, as has been reported previously (Wang and Below, 1995, 1998).

Phytate, which forms complexes with divalent cations, has been found to hinder human Zn and Fe absorption during digestion and thus has been labeled an “anti-nutrient.” It may serve a number of valuable functions, however, including roles as an anti-oxidant and anti-cancer agent (Raboy, 2009). Phytate is also the major repository of grain P, and variation in P supply to the developing seed is the major determinant of net seed phytate accumulation (Raboy, 1997, 2009; Cakmak et al., 2010). To our knowledge, no published studies have explicitly looked at how phytate is affected by CO_2_ concentration. Elevated CO_2_ has been found to have a much larger negative impact on Zn and Fe concentrations than on P in wheat (Loladze, 2002; Cakmak et al., 2010). Several studies (e.g., Fangmeier et al., 1999; Högy and Fangmeier, 2008) have observed that P increases slightly with CO_2_ concentration, and because the majority of P is tied up in phytate, this may cause increases in grain phytate concentrations as atmospheric CO_2_ rises. As a result, bioavailable Zn and Fe–Zn and Fe not bound to phytate – is expected to decrease even further (Loladze, 2002).

Nonetheless, we did not observe such trends in macro- and micronutrient concentrations in this study. The mechanism behind these contrasting results is not clear, although the environmental conditions and nutrient solution in which the plants were grown likely had some role. The modeled data demonstrated only a small negative impact of CO_2_ concentration on bioavailable Zn concentrations (Table 4), which was unexpected. Indeed, the grain from ${\text{NO}}_{\text{3}}^{-}$-supplied plants actually showed a slight increase in bioavailable Zn between ambient and elevated CO_2_. These results combined with the differences in grain bioavailable Zn between ${\text{NH}}_{4}^{+}$ and ${\text{NO}}_{\text{3}}^{-}$-supplied plants demonstrates that N form may differentially affect the nutritional status of this important nutrient, especially in less developed countries that might be more dependent on phytate-rich grains for their Zn nutrition (Table 3). The milling process removes some, if not most, of the phytate and grain mineral content with the bran fraction of the grain (Guttieri et al., 2006). Regardless, with over 50% of the human population suffering from Zn deficiencies, even small increases in bioavailable Zn would be beneficial (Loladze, 2002). This modeling exercise, however, is not a prediction of how increasing CO_2_ will affect wheat nutrition so much as illustrates that N source may mediate, to some extent, the effects of CO_2_ on phytate and bioavailable Zn, and that N source will become an even more important agricultural consideration in the future.

In summary, both CO_2_ concentration and N form strongly affect biomass and yield in hydroponically grown wheat, as well as nutrient concentrations in above- and belowground tissues. Interactions among plant nutrient concentrations, CO_2_ concentrations, and N form are complex and non-linear. The impact of N form and CO_2_ concentration on the mechanisms affecting nutrient accumulation and distribution requires further research and extension to more realistic and agriculturally relevant growing conditions found in greenhouse and field studies. Of course, in greenhouse and field studies, control of N source is limited and control of atmospheric CO_2_ concentration is expensive. The effects of CO_2_ and N form on agriculture and human nutrition observed here are interesting and suggest a new area of research on mitigating the effects of climate change on agriculture. The supply of fertilizers (e.g., urea, NH_4_NO_3_, anhydrous NH_3_, organic amendments) or addition of nitrification inhibitors that increase the amount of available ${\text{NH}}_{4}^{+}$ may have beneficial effects for human nutrition, particularly in regards to micronutrient deficiencies such as Zn and Fe that currently affect billions of people worldwide. In the face of the potentially negative consequences of climate change on agriculture, all avenues of mitigation must be examined, and even small improvements may prove worthwhile.

## Conflict of Interest Statement

The authors declare that the research was conducted in the absence of any commercial or financial relationships that could be construed as a potential conflict of interest.
